# Correlation of Adiponectin Gene Polymorphisms *rs266729* and *rs3774261* With Risk of Nonalcoholic Fatty Liver Disease: A Systematic Review and Meta-Analysis

**DOI:** 10.3389/fendo.2022.798417

**Published:** 2022-03-23

**Authors:** Yong-Tian Zheng, Tian-Mei Xiao, Chan-Xian Wu, Jin-Yan Cheng, Le-Yu Li

**Affiliations:** Department of Endocrinology, Zhongshan Hospital of Chinese Traditional Medicine, Zhongshan, China

**Keywords:** adiponectin, polymorphism, nonalcoholic fatty liver disease, system review, meta-analysis

## Abstract

**Background:**

Increasing evidence has suggested an association of adiponectin gene polymorphisms rs1501299, rs2241766, rs266729 and rs3774261 with risk of nonalcoholic fatty liver disease (NAFLD). This correlation has been extensively meta-analyzed for the first two polymorphisms, but not the second two.

**Methods:**

The PubMed, EMBASE, Google Scholar, and China National Knowledge Infrastructure databases were searched for relevant literature. Odds ratios (ORs) and 95% confidence intervals (CIs) were calculated.

**Results:**

A total of 10 case-control studies on rs266729 (2,619 cases and 1,962 controls) and 3 case-control studies on rs3774261 (562 cases and 793 controls) were included. Meta-analysis showed that rs266729 was associated with significantly higher NAFLD risk based on the following five models: allelic, OR 1.72, 95% CI 1.34-2.21, *P* < 0.001; recessive, OR 2.35, 95% CI 1.86-2.95, *P* < 0.001; dominant, OR 1.84, 95% CI 1.34-2.53, *P* < 0.001; homozygous, OR 2.69, 95% CI 1.84-3.92, *P* < 0.001; and heterozygous, OR 1.72, 95% CI 1.28-2.32, *P* < 0.001. This association between rs266729 and NAFLD risk remained significant for all five models among studies with Asian, Chinese and Caucasian samples. The rs2241766 polymorphism was associated with significantly higher NAFLD risk according to the recessive model (OR 1.87, 95% CI 1.15-3.04, *P* = 0.01).

**Conclusion:**

Polymorphisms rs266729 and rs3774261 in the adiponectin gene may be risk factors for NAFLD. These findings may pave the way for novel therapeutic strategies, but they should be verified in large, well-designed studies.

## Introduction

Nonalcoholic fatty liver disease (NAFLD), also known as metabolism-associated fatty liver disease (1), is rapidly becoming the most common liver disease worldwide. The primary characteristic of NAFLD is hepatocellular macrovesicular steatosis. NAFLD can progress to hepatic injury, which can range from simple steatosis or nonalcoholic steatohepatitis (NASH), to fibrosis, cirrhosis, and even hepatocellular carcinoma or end-stage liver disease (2–6). NAFLD and its progression have been linked to diet (7), insulin resistance (8, 9), lipotoxicity (10), inflammation (11, 12), genetic predisposition and increases in compounds produced by gut microbes (13, 14). Genetic factors, for example, can alter hepatic lipid metabolism. In this way, NAFLD is a complex metabolic state to which lifestyle and genetic factors contribute (15, 16).

Adiponectin is a protein specific to adipose tissue that regulates insulin sensitivity, glucose homeostasis, and lipid metabolism (17). Decreased levels of adiponectin in plasma are associated with NAFLD as well as obesity, type 2 diabetes, and coronary artery disease (18, 19). Adiponectin is encoded by the 16-kb *AMP1* gene on human chromosome 3q27, and it consists of three exons and two introns. Genetic and epigenetic changes in the adiponectin gene may reduce adiponectin levels in plasma and dysregulate hepatic lipid metabolism, which may help explain differences in NAFLD risk among individuals (20, 21). Thus, single-nucleotide polymorphisms (SNPs) in the adiponectin gene may alter levels of the protein in circulation, in turn affecting lipid metabolism and NAFLD risk.

The two adiponectin SNPs most thoroughly investigated for their association with NAFLD risk are rs2241766, which leads to genomic mutation T45G, and rs1501299, which leads to mutation G276T (22–31). Indeed, these two associations have been extensively reviewed and meta-analyzed (32–35). In contrast, much less is known about potential associations of the polymorphisms rs266729 (-11377C>G) and rs3774261 with NAFLD risk (36–47).

Thus, we meta-analyzed here the relevant literature on potential associations of rs266729 and rs3774261 with NAFLD risk.

## Material and Methods

### Search Strategy

The PubMed, EMBASE, Google Scholar, Web of Science and China National Knowledge Infrastructure (CNKI) databases were searched up to October 20, 2021 without language restrictions using the following search terms: (a) *adiponectin, ADIPOQ, APMI, -11377, -11377C>G, rs266729* and *rs3774261*; (b) those seven terms in combination with *polymorphisms, SNP, variant, variants, variation, genotype, genetic* or *mutation;* and (c) all of the above terms in combination with nonalcoholic fatty liver disease or NAFLD. Only studies involving humans were considered. Reference lists in original and review articles were searched manually to identify additional studies. In the case of multiple studies involving overlapping samples, only the largest study was retained.

### Inclusion and Exclusion Criteria of the Studies

Studies were included if they met the following criteria: (a) studies had a case-control design to assess the association of adiponectin rs266729 or rs3774261 with NAFLD risk; (b) all patients were diagnosed with NAFLD based on the following diagnostic criteria: abnormal levels of aspartate aminotransferase and alanine aminotransferase persisting for at least 6 months, or evidence of fatty liver based on ultrasonography and/or evidence of diffuse fatty liver based on other imaging examinations, or liver histology; (c) the full text was available and it reported genotype frequencies in cases and controls, or sufficient data to estimate odds ratios (ORs) and 95% confidence intervals (CIs).

Studies were excluded if they: (a) were not a case-control study; (b) did not report precise genotypes; (c) were duplicate publications of data from the same study; (d) were meta-analyses, letters, reviews, or editorial articles; (e) investigated other polymorphisms of adiponectin.

### Data Extraction

Two authors (YTZ and LYL) independently selected eligible studies and extracted the following data: first author’s name, year of publication, ethnicity, country, sample size, type of controls, genotyping method, genotype distribution, *P* value for Hardy-Weinberg equilibrium among controls, and matched parameters.

### Assessment of Methodological Quality

The quality of included studies was assessed independently by two investigators (YTZ and LYL) using the Newcastle–Ottawa Scale (48). Scores of 0-4 were considered to indicate poor methodological quality; scores of 5-9, high quality (49). Any disagreements about scoring were resolved through comprehensive reassessment by the other authors. Only high-quality studies were included in the meta-analysis.

### Statistical Analysis

The strength of association of rs266729 and rs3774261 with NAFLD risk was calculated in terms of unadjusted ORs with 95% CIs based on genotype frequencies in cases and controls. The significance of pooled ORs was determined using the Z test, with P < 0.05 defined as significant. Meta-analysis was conducted using a fixed-effect model when P > 0.10 for the Q test, indicating lack of heterogeneity among studies; otherwise, meta-analysis was conducted using a random-effect model. All statistical tests for meta-analyses were performed using Review Manager 5.3 (Cochrane Collaboration). Publication bias was assessed using Begg’s funnel plot and Egger’s weighted regression in Stata 12.0 (Stata Corp, College Station, TX, USA), with P < 0.05 considered statistically significant.

## Results

### Characteristics of Primary Studies

**Figure 1:**
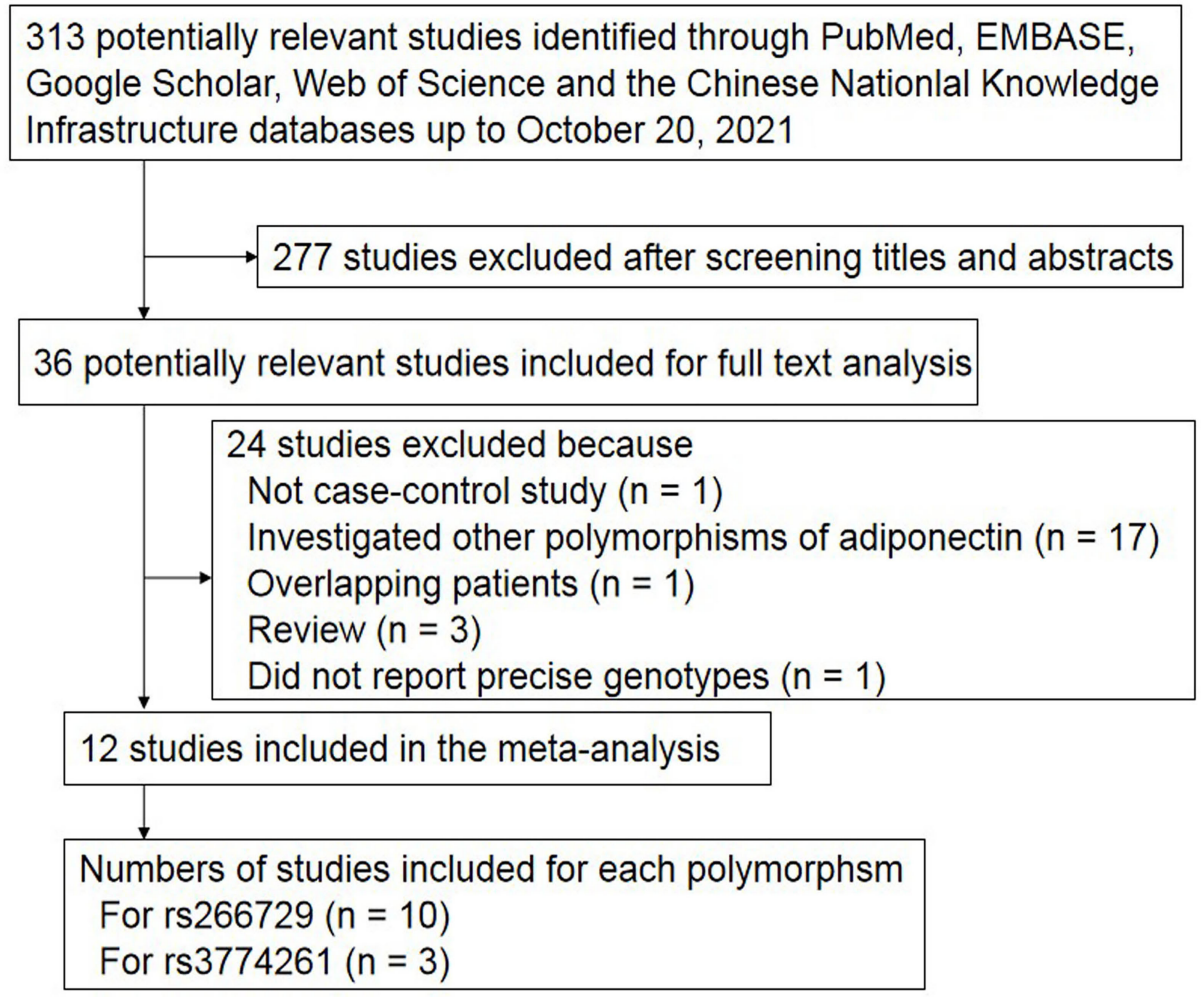
Flowchart of study selection.

The search strategy retrieved 313 potentially relevant studies, 277 of which were excluded on the basis of titles and abstracts (**Figure 1**). Another 17 studies were excluded because they investigated other polymorphisms of the adiponectin gene, one study was excluded because it enrolled only cases (50), three studies were excluded because they were review articles (51–53), and one study was excluded because it did not report precise genotypes (30). Two publications were based on the same participants, so they were considered as one study (38, 54). Ultimately, 12 case-control studies (36–47) were included in the meta-analysis (**Table 1**).

**Table 1:** Characteristics of the included studies and genotype distributions.

First author	Year	Ethnicity	Country	Sample size		Genotyping method	*P* for HWE	Source of controls	No. of cases			No. of controls			NOS score	Matched parameters
				Cases	Controls											
**rs266729**									**CC**	**CG**	**GG**	**CC**	**CG**	**GG**		
Gupta (36)	2012	Asian	India	137	250	PCR	0.003	HB	77	53	7	156	92	2	7	Age, Sex, BMI
Hashemi (37)	2013	Asian	India	83	93	Tetra ARMS-PCR	0.107	PB	27	53	3	49	41	3	6	Undetermined
Ye (38)	2014	Asian	China	130	130	PCR-RFLP	0.458	HB	77	47	6	83	40	7	7	Age, Sex
Hsieh (39)	2015	Asian	Taiwan, China	350	209	TaqMan	0.545	T2DM without NAFLD	175	126	49	113	79	17	7	Age, Sex
Cheng (40)	2015	Asian	China	338	280	PCR	0.715	HB	158	149	31	164	102	14	7	Age, Sex, Height
Zhang (41)	2016	Asian	China	302	310	PCR-RFLP	0.619	HB	152	126	24	197	102	11	7	Age, Sex, Drinking, Smoking
Zhang (42)	2016	Asian	China	600	200	PCR-RFLP	<0.001	HB	180	202	218	143	28	29	7	Age, Sex, Ethnicity, Birthplace
Du (43)	2016	Asian	China	493	304	PCR	0.068	HB	278	175	40	219	73	12	7	Age, Sex
Mahmoud (44)	2019	Caucasian	Egypt	100	100	PCR	0.539	HB	38	44	18	47	45	8	7	Age, Sex
Hasan (45)	2021	Caucasian	Egypt	86	86	PCR-RFLP	0.236	HB	28	53	5	44	38	4	6	Age
**rs3774261**									**AA**	**AG**	**GG**	**AA**	**AG**	**GG**		
Zhang (46)	2012	Asian	China	119	350	PCR-RFLP	0.808	PB	41	50	28	107	171	72	7	Sex
Li (47)	2015	Asian	China	357	357	PCR-RFLP	0.044	HB	48	179	130	131	155	71	7	Age, Sex
Hasan (45)	2021	Caucasian	Egypt	86	86	PCR-RFLP	0.954	HB	11	55	20	39	38	9	6	Age

T2DM, diabetes mellitus type 2; NAFLD, nonalcoholic fatty liver disease risk; ARMS, amplification refractory mutation system; PCR, polymerase chain reaction; RFLP, restriction fragment length polymorphism; HB, hospital-based; PB, population-based; BMI, body mass index; HWE, Hardy-Weinberg equilibrium; NOS, Newcastle–Ottawa Scale.

Ten studies (36–45) focused on rs266729 and three (45–47) on rs3774261. The distribution of genotypes in controls was consistent with Hardy-Weinberg equilibrium in all but three studies (36, 42, 47). The mean Newcastle-Ottawa score for the 12 studies was 6.83 (range, 6-7). Thus the overall quality of the included studies was adequate.

### Quantitative Data Synthesis

#### rs266729 and NAFLD Risk

**Figure 2:**
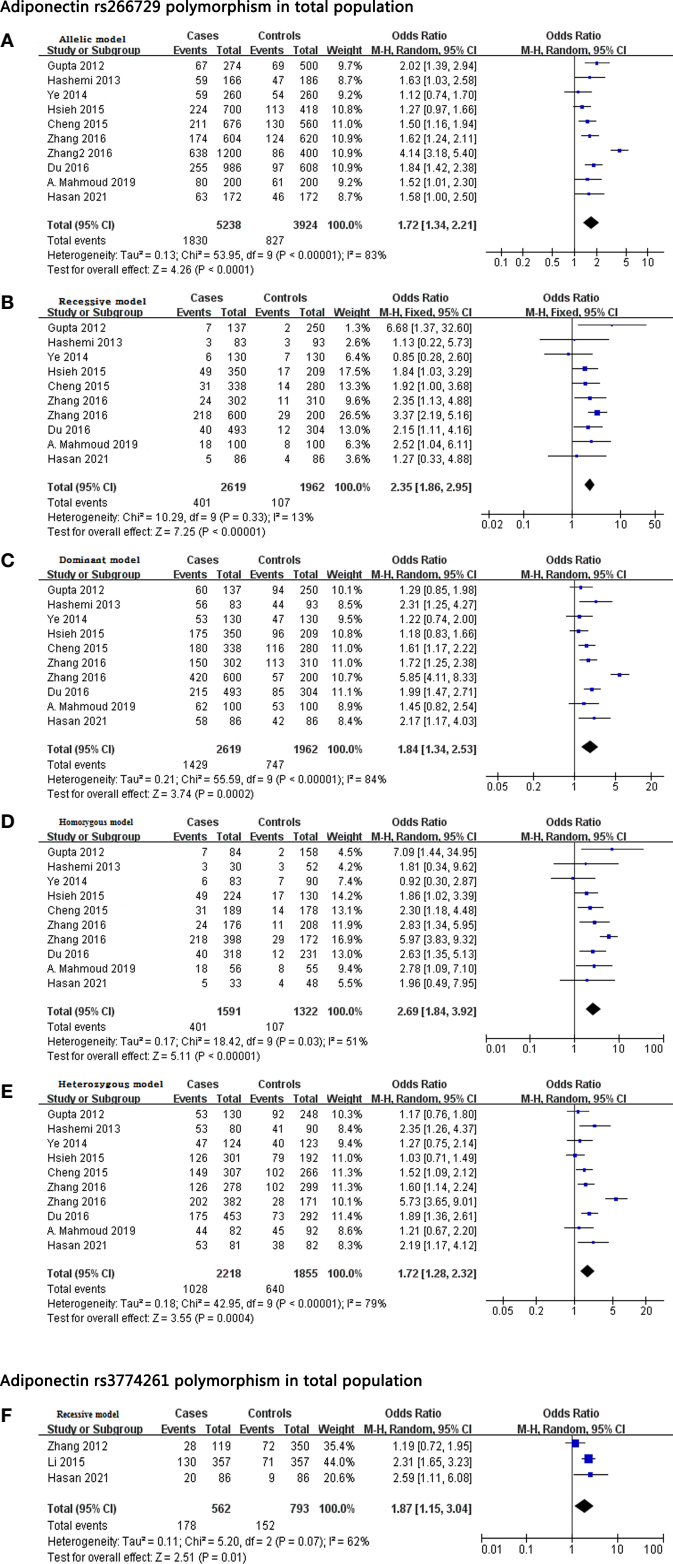
**(A–E)** Forest plot showing the relationship between rs266729 polymorphism and NAFLD risk in the total population according to different genetic models: **(A)** allelic (G-allele vs. C-allele), **(B)** recessive (GG vs. CG + CC), **(C)** dominant (CG + GG vs. CC), **(D)** homozygous (GG vs. CC), or **(E)** heterozygous (CG vs. CC). **(F)** Forest plot showing the relationship between rs3774261 polymorphism and NAFLD risk in the total population according to the recessive model (GG vs. AG + AA). CI, confidence interval; *df*, degree of freedom; M-H, Mantel-Haenszel; NAFLD, nonalcoholic fatty liver disease.

Meta-analysis of data from 2,619 cases and 1,962 controls indicated that rs266729 was associated with significantly increased NAFLD risk according to the following five models: allelic, OR 1.72, 95% CI 1.34-2.21, *P* < 0.001; recessive, OR 2.35, 95% CI 1.86-2.95, *P* < 0.001; dominant, OR 1.84, 95% CI 1.34-2.53, *P* < 0.001; homozygous, OR 2.69, 95% CI 1.84-3.92, *P* < 0.001; and heterozygous, OR 1.72, 95% CI 1.28-2.32, *P* < 0.001 (**Table 2** and **Figures 2A–E**).

**Table 2:** Meta-analysis of associations of rs266729 or rs3774261 with risk of nonalcoholic fatty liver disease.

Genetic model	OR [95% CI]	Z (*P* value)	Heterogeneity of study design			Meta-analysis model
			χ^2^	df (*P* value)	I^2^ (%)	
***Adiponectin rs266729 polymorphism***						
Adiponectin rs266729 polymorphism in total population from 10 case control studies (36–45) (2,619 cases and 1,962 controls)						
Allelic model (G-allele vs. C-allele)	1.72 [1.34, 2.21]	4.26 (<0.001)	53.95	9 (<0.001)	83	Random
Recessive model (GG vs. CG + CC)	2.35 [1.86, 2.95]	7.25 (<0.001)	10.29	9 (0.33)	13	Fixed
Dominant model (CG + GG vs. CC)	1.84 [1.34, 2.53]	3.74 (<0.001)	55.59	9 (<0.001)	84	Random
Homozygous model (GG vs. CC)	2.69 [1.84, 3.92]	5.11 (<0.001)	18.42	9 (0.03)	51	Random
Heterozygous model (CG vs. CC)	1.72 [1.28, 2.32]	3.55 (<0.001)	42.95	9 (<0.001)	79	Random
Adiponectin rs266729 polymorphism in Asian population from 8 case-control studies (36–43) (2,433 cases and 1,776 controls)						
Allelic model (G-allele vs. C-allele)	1.76 [1.31, 2.37]	3.75 (<0.001)	53.01	7 (<0.001)	87	Random
Recessive model (GG vs. CG + CC)	2.38 [1.86, 3.03]	6.98 (<0.001)	9.48	7 (0.22)	26	Fixed
Dominant model (CG + GG vs. CC)	1.85 [1.28, 2.69]	3.26 (0.001)	54.56	7 (<0.001)	87	Random
Homozygous model (GG vs. CC)	2.70 [1.73, 4.23]	4.35 (0.001)	18.00	7 (0.01)	61	Random
Heterozygous model (CG vs. CC)	1.75 [1.23, 2.47]	3.15 (0.002)	41.11	7 (<0.001)	83	Random
Adiponectin rs266729 polymorphism in Chinese population from 6 case-control studies (38–43) (2,213 cases and 1,433 controls)						
Allelic model (G-allele vs. C-allele)	1.74 [1.20, 2.52]	2.94 (0.003)	52.49	5 (<0.001)	90	Random
Recessive model (GG vs. CG + CC)	2.35 [1.83, 3.01]	6.72 (<0.001)	7.01	5 (0.22)	29	Fixed
Dominant model (CG + GG vs. CC)	1.91 [1.21, 3.00]	2.78 (0.005)	50.92	5 (<0.001)	90	Random
Homozygous model (GG vs. CC)	2.58 [1.57, 4.24]	3.75 (<0.001)	16.55	5 (0.005)	70	Random
Heterozygous model (CG vs. CC)	1.79 [1.18, 2.73]	2.71 (0.007)	37.20	5 (<0.001)	87	Random
Adiponectin rs266729 polymorphism in Caucasian population from 2 case-control studies (44, 45) (186 cases and 186 controls)						
Allelic model (G-allele vs. C-allele)	1.55 [1.14, 2.10]	2.79 (0.005)	0.02	1 (0.90)	0	Fixed
Recessive model (GG vs. CG + CC)	2.07 [0.99, 4.30]	1.94 (0.05)	0.70	1 (0.40)	0	Fixed
Dominant model (CG + GG vs. CC)	1.74 [1.15, 2.63]	2.61 (0.009)	0.90	1 (0.34)	0	Fixed
Homozygous model (GG vs. CC)	2.51 [1.16, 5.44]	2.33 (0.02)	0.16	1 (0.68)	0	Fixed
Heterozygous model (CG vs. CC)	1.60 [1.04, 2.46]	2.14 (0.03)	1.80	1 (0.18)	45	Fixed
***Adiponectin rs3774261 polymorphism***						
Adiponectin rs3774261 polymorphism in total population from 3 case-control studies (45–47) (562 cases and 793 controls)						
Allelic model (G-allele vs. A-allele)	1.76 [0.98, 3.18]	1.86 (0.06)	22.72	2 (<0.001)	91	Random
Recessive model (GG vs. AG + AA)	1.87 [1.15, 3.04]	2.51 (0.01)	5.20	2 (0.07)	62	Random
Dominant model (AG + GG vs. AA)	2.55 [0.81, 7.98]	1.60 (0.11)	31.99	2 (<0.001)	94	Random
Homozygous model (GG vs. AA)	3.29 [0.97, 11.15]	1.91 (0.06)	22.63	2 (<0.001)	91	Random
Heterozygous model (AG vs. AA)	2.25 [0.75, 6.76]	1.45 (0.15)	26.19	2 (<0.001)	92	Random
Adiponectin rs3774261 polymorphism in Chinese population from 2 case-control studies (46, 47) (476 cases and 707 controls)						
Allelic model (G-allele vs. A-allele)	1.49 [0.66, 3.35]	0.97 (0.33)	19.79	1 (<0.001)	95	Random
Recessive model (GG vs. AG + AA)	1.70 [0.89, 3.25]	1.60 (0.11)	4.69	1 (0.03)	79	Random
Dominant model (AG + GG vs. AA)	1.78 [0.41, 7.68]	0.77 (0.44)	25.73	1 (<0.001)	96	Random
Homozygous model (GG vs. AA)	2.28 [0.48, 10.85]	1.03 (0.30)	19.02	1 (<0.001)	95	Random
Heterozygous model (AG vs. AA)	1.56 [0.39, 6.27]	0.63 (0.53)	20.11	1 (<0.001)	95	Random

OR, odds ratio; 95% CI, 95% confidence interval.

This association remained significant when we meta-analyzed only the eight studies involving 2,433 Asian cases and 1,776 Asian controls (36–43). Again, significance was obtained with all five models: allelic, OR 1.76, 95% CI 1.31-2.37, *P* < 0.001; recessive, OR 2.38, 95% CI 1.86-3.03, *P* < 0.001; dominant, OR 1.85, 95% CI 1.28-2.69, *P* = 0.001; homozygous, OR 2.70, 95% CI 1.73-4.23, *P* = 0.001; and heterozygous, OR 1.75, 95% CI 1.23-2.47, *P* = 0.002 (**Table 2** **)**.

Next, this association remained significant when we meta-analyzed only the eight studies involving 2,213 Chinese cases and 1,433 Chinese controls (38–43). Again, significance was obtained with all five models: allelic, OR 1.74, 95% CI 1.20-2.52, *P* = 0.003; recessive, OR 2.35, 95% CI 1.83-3.01, *P* < 0.001; dominant, OR 1.91, 95% CI 1.21-3.00, *P* = 0.005; homozygous, OR 2.58, 95% CI 1.57-4.24, *P* < 0.001; and heterozygous, OR 1.79, 95% CI 1.18-2.73, *P* = 0.007 (**Table 2** **)**.

Lastly, this association remained significant when we meta-analyzed only the eight studies involving 186 Caucasian cases and 186 Caucasian controls (44, 45). Again, significance was obtained with all five models: allelic, OR 1.55, 95% CI 1.14-2.10, *P* = 0.005; recessive, OR 2.07, 95% CI 0.99-4.30, *P* = 0.05; dominant, OR 1.74, 95% CI 1.15-2.63, *P* = 0.009; homozygous, OR 2.51, 95% CI 1.16-5.44, *P* = 0.02; and heterozygous, OR 1.60, 95% CI 1.04-2.46, *P* = 0.03 (**Table 2** **)**.

#### rs3774261 and NAFLD risk

Meta-analysis of three studies (45–47) involving 562 cases and 793 controls showed that rs3774261 was associated with significantly increased NAFLD risk according to the recessive model (OR 1.87, 95% CI 1.15-3.04, *P* = 0.01; **Table 2** and **Figure 2F** **)**. But this association could not be found in the Chinese population (**Table 2** **)**.

### Sensitivity Analysis

To assess the reliability of the outcomes in the meta-analysis, we repeated the meta-analysis after excluding, one by one, three studies in which the *P* value associated with Hardy-Weinberg equilibrium was less than 0.05 (36, 42, 47).

After excluding the study by Gupta et al. (36), the results did not differ substantially either in total or in Asian population for rs266729 polymorphism (**Supplementary Table S1**).

After excluding the study by Zhang et al. (42), the results did not differ substantially in total, Asian or Chinese population for rs266729 polymorphism (**Supplementary Table S2**).

After excluding the study by Li et al. (47), the results were altered in recessive model in total population for rs3774261 polymorphism (**Supplementary Table S3**). Therefore, the results for rs3774261 polymorphism should be interpreted with caution.

### Publication Bias

**Figure 3:**
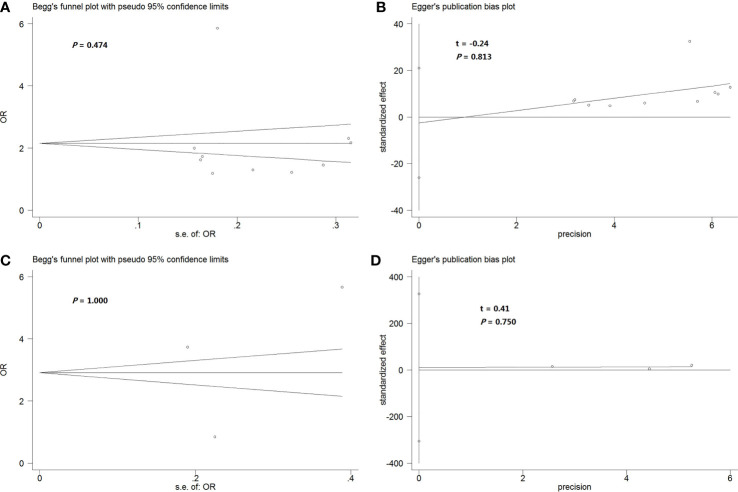
**(A, B)** Begg’s funnel plot **(A)** and Egger’s test **(B)** to assess publication bias in the meta-analysis of the association between rs266729 polymorphism and NAFLD risk in the total population according to the dominant genetic model. **(C, D)** Begg’s funnel plot **(C)** and Egger’s test **(D)** to assess publication bias in the meta-analysis of the association between rs3774261polymorphism and NAFLD risk in the total population according to the dominant genetic model. NAFLD, nonalcoholic fatty liver disease; OR, odds ratio.

Begg’s funnel plot and Egger’s test were performed to detect potential publication bias in our meta-analysis. Funnel plots showed no obvious asymmetry in the dominant model of the rs266729 polymorphism (**Figure 3A**), and the result for Egger’s test was not significant (**Figure 3B**). Similar results were obtained with the dominant model of the rs3774261 polymorphism (**Figures 3C, D**).

## Discussion

The physiological roles of adiponectin remain unclear, but it has been associated with obesity, insulin resistance, type 2 diabetes, atherosclerosis, hypertension, coronary artery disease, various inflammatory diseases, metabolic syndrome and NAFLD (18, 19, 55, 56). In fact, high levels of adiponectin may protect against NAFLD (56), perhaps by activating AMPK and peroxisome proliferator-activated receptor γ to improve insulin sensitivity, reduce fatty acid synthesis and enhance fatty acid oxidation (57). Here we provide additional evidence that adiponectin levels may influence onset of NAFLD by demonstrating associations between two SNPs in the adiponectin gene and risk of the disorder.

We found that rs266729 was significantly related to elevated NAFLD risk across all ethnic groups examined, as well as specifically in Asian, Chinese and Caucasian populations. Consistent with our findings, a previous meta-analysis (35) of three case-control studies (30, 36, 37) suggested a similar association among Asians. We included two of those case-control studies in the present meta-analysis but not one (30) because it did not report precise genotypes. Another Chinese study (43) reported an association between rs266729 and elevated NAFLD risk, as well as elevated risk of coronary artery disease among NAFLD patients. Our results extend the findings of a previous study linking rs266729 to elevated NAFLD risk in a southeastern Iranian population (37). However, our results contrast with a study (38) that failed to link rs266729 to NAFLD risk among Han Chinese. The relatively large sample in our meta-analysis may make our findings more reliable.

We also found that rs3774261 was significantly related to elevated NAFLD risk across all ethnic groups examined. The fact that our meta-analysis contained only three case-control studies involving 562 cases and 793 controls emphasizes the need for further research. Indeed, further research is needed into the potential association of the adiponectin SNPs rs17300539 (G–11391A) (24, 58) and rs822393 (42) and risk of NAFLD. We were unable to include those SNPs in our meta-analysis because of the limited data available.

Our results should be interpreted with caution in light of several limitations. First, the controls in one study (39) had diabetes mellitus type 2, so they may not be comparable to healthy controls in other studies. Second, the *P* value associated with Hardy-Weinberg equilibrium was < 0.001 in three studies (36, 42, 47), suggesting a lack of generalizability to the broader population. Nevertheless, excluding each of those studies one at a time did not substantially alter the meta-analysis. Third, the robustness of our meta-analysis may be reduced by the fact that studies used genotyping methods differing in sensitivity and specificity, and by confounding due to sex, age, insulin resistance, family history of type 2 diabetes, obesity, coronary artery disease, hypertension and metabolic syndrome. We were unable to account for those factors in our meta-analysis because the original studies either did not report their frequencies or they aggregated the factors in different ways.

## Conclusion

The available evidence suggests that SNPs rs266729 and rs3774261 in the adiponectin gene are risk factors for NAFLD. If our results can be verified in large, well-designed studies, they may help pave the way for novel therapeutic strategies.

## Data Availability Statement

The original contributions presented in the study are included in the article/**Supplementary Material**. Further inquiries can be directed to the corresponding author.

## Author Contributions

Designed the study: L-YL and Y-TZ. Searched databases and collected full-text papers: T-MX and C-XW. Extracted and analyzed the data: L-YL and Y-TZ. Statistical analyses: J-YC. Wrote the manuscript: Y-TZ. All authors reviewed the manuscript. All authors contributed to the article and approved the submitted version.

## Conflict of Interest

The authors declare that the research was conducted in the absence of any commercial or financial relationships that could be construed as a potential conflict of interest.

## Publisher’s Note

All claims expressed in this article are solely those of the authors and do not necessarily represent those of their affiliated organizations, or those of the publisher, the editors and the reviewers. Any product that may be evaluated in this article, or claim that may be made by its manufacturer, is not guaranteed or endorsed by the publisher.
