# Supplementary material for: Correlation of Adiponectin Gene Polymorphisms rs266729 and rs3774261 With Risk of Nonalcoholic Fatty Liver Disease: A Systematic Review and Meta-Analysis
Source: Front Endocrinol (Lausanne). 2022 Mar 23;13:798417. doi: 10.3389/fendo.2022.798417 (PMC8983824; doi:10.3389/fendo.2022.798417)
Supplement: Supplementary file 1 [file Table_1.docx]

**Supplementary Table S1.** Meta-analysis of associations of rs266729 with risk of nonalcoholic fatty liver disease after excluding the study by Gupta et al. [36].

| Genetic model | OR [95% CI] | Z (*P* value) | Heterogeneity of study design | | | Meta-analysis model |
| --- | --- | --- | --- | --- | --- | --- |
|  |  |  | χ^2^ | df (*P* value) | I^2^ (%) |  |
| ***Adiponectin rs266729 polymorphism*** | | | | | | |
| Adiponectin rs266729 polymorphism in total population from 9 case control studies [37-45] (2,482 cases and 1,712 controls) | | | | | | |
| Allelic model (G-allele vs. C-allele) | 1.69 [1.29, 2.22] | 3.76 (<0.001) | 53.50 | 8 (<0.001) | 85 | Random |
| Recessive model (GG vs. CG + CC) | 2.29 [1.81, 2.89] | 6.96 (<0.001) | 8.52 | 8 (0.38) | 6 | Fixed |
| Dominant model (CG + GG vs. CC) | 1.91 [1.36, 2.70] | 3.69 (<0.001) | 52.40 | 8 (<0.001) | 85 | Random |
| Homozygous model (GG vs. CC) | 2.56 [1.74, 3.78] | 4.74 (<0.001) | 17.29 | 8 (0.03) | 54 | Random |
| Heterozygous model (CG vs. CC) | 1.80 [1.30, 2.49] | 3.56 (<0.001) | 40.02 | 8 (<0.001) | 80 | Random |
| Adiponectin rs266729 polymorphism in Asian population from 7 case-control studies [37-43] (2,433 cases and 1,776 controls) | | | | | | |
| Allelic model (G-allele vs. C-allele) | 1.73 [1.24, 2.40] | 3.23 (0.001) | 52.68 | 6 (<0.001) | 89 | Random |
| Recessive model (GG vs. CG + CC) | 2.31 [1.81, 2.96] | 6.68 (<0.001) | 7.75 | 6 (0.26) | 23 | Fixed |
| Dominant model (CG + GG vs. CC) | 1.95 [1.29, 2.94] | 3.20 (0.001) | 51.21 | 6 (<0.001) | 88 | Random |
| Homozygous model (GG vs. CC) | 2.54 [1.59, 4.05] | 3.92 (<0.001) | 16.92 | 6 (0.01) | 65 | Random |
| Heterozygous model (CG vs. CC) | 1.85 [1.26, 2.71] | 3.16 (0.002) | 38.08 | 6 (<0.001) | 84 | Random |

**Abbreviations**: OR, odds ratio; 95% CI, 95% confidence interval.
