# Supplementary material for: Correlation of Adiponectin Gene Polymorphisms rs266729 and rs3774261 With Risk of Nonalcoholic Fatty Liver Disease: A Systematic Review and Meta-Analysis
Source: Front Endocrinol (Lausanne). 2022 Mar 23;13:798417. doi: 10.3389/fendo.2022.798417 (PMC8983824; doi:10.3389/fendo.2022.798417)
Supplement: Supplementary file 2 [file Table_2.docx]

**Supplementary Table S2.** Meta-analysis of associations of rs266729 with risk of nonalcoholic fatty liver disease after excluding the study by Zhang et al. [42].

| Genetic model | OR [95% CI] | Z (*P* value) | Heterogeneity of study design | | | Meta-analysis model |
| --- | --- | --- | --- | --- | --- | --- |
|  |  |  | χ^2^ | df (*P* value) | I^2^ (%) |  |
| ***Adiponectin rs266729 polymorphism*** | | | | | | |
| Adiponectin rs266729 polymorphism in total population from 9 case control studies [36-41, 43-45] (2,019 cases and 1,762 controls) | | | | | | |
| Allelic model (G-allele vs. C-allele) | 1.55 [1.39, 1.73] | 8.04 (<0.001) | 8.26 | 8 (0.41) | 3 | Fixed |
| Recessive model (GG vs. CG + CC) | 1.98 [1.50, 2.61] | 4.84 (<0.001) | 5.96 | 8 (0.65) | 0 | Fixed |
| Dominant model (CG + GG vs. CC) | 1.59 [1.39, 1.82] | 6.88 (<0.001) | 9.78 | 8 (0.28) | 18 | Fixed |
| Homozygous model (GG vs. CC) | 2.29 [1.73, 3.04] | 5.74 (<0.001) | 5.61 | 8 (0.69) | 0 | Fixed |
| Heterozygous model (CG vs. CC) | 1.49 [1.30, 1.71] | 5.65 (<0.001) | 11.64 | 8 (0.17) | 31 | Fixed |
| Adiponectin rs266729 polymorphism in Asian population from 8 case-control studies [36-41, 43] (1,833 cases and 1,576 controls) | | | | | | |
| Allelic model (G-allele vs. C-allele) | 1.55 [1.39, 1.74] | 7.54 (<0.001) | 8.52 | 6 (0.22) | 27 | Fixed |
| Recessive model (GG vs. CG + CC) | 1.96 [1.46, 2.65] | 4.44 (<0.001) | 5.24 | 6 (0.51) | 0 | Fixed |
| Dominant model (CG + GG vs. CC) | 1.58 [1.37, 1.81] | 6.38 (<0.001) | 8.68 | 6 (0.19) | 31 | Fixed |
| Homozygous model (GG vs. CC) | 2.26 [1.67, 3.07] | 5.26 (<0.001) | 5.38 | 6 (0.50) | 0 | Fixed |
| Heterozygous model (CG vs. CC) | 1.48 [1.28, 1.71] | 5.24 (<0.001) | 9.72 | 6 (0.14) | 38 | Fixed |
| Adiponectin rs266729 polymorphism in Chinese population from 5 case-control studies [38-43] (1,613 cases and 1,233 controls) | | | | | | |
| Allelic model (G-allele vs. C-allele) | 1.51 [1.33, 1.71] | 6.45 (<0.001) | 6.03 | 4 (0.20) | 34 | Fixed |
| Recessive model (GG vs. CG + CC) | 1.90 [1.39, 2.59] | 4.06 (<0.001) | 2.45 | 4 (0.65) | 0 | Fixed |
| Dominant model (CG + GG vs. CC) | 1.58 [1.36 1.84] | 5.85 (<0.001) | 6.35 | 4 (0.17) | 37 | Fixed |
| Homozygous model (GG vs. CC) | 2.17 [1.58, 2.97] | 4.79 (<0.001) | 3.26 | 4 (0.52) | 0 | Fixed |
| Heterozygous model (CG vs. CC) | 1.48 [1.26, 1.74] | 4.78 (<0.001) | 6.46 | 4 (0.17) | 38 | Fixed |

**Abbreviations**: OR, odds ratio; 95% CI, 95% confidence interval.
