# Supplementary material for: Correlation of Adiponectin Gene Polymorphisms rs266729 and rs3774261 With Risk of Nonalcoholic Fatty Liver Disease: A Systematic Review and Meta-Analysis
Source: Front Endocrinol (Lausanne). 2022 Mar 23;13:798417. doi: 10.3389/fendo.2022.798417 (PMC8983824; doi:10.3389/fendo.2022.798417)
Supplement: Supplementary file 3 [file Table_3.docx]

**Supplementary Table S3**. Meta-analysis of associations of rs3774261 with risk of nonalcoholic fatty liver disease after excluding the study by Li et al. [47].

| Genetic model | OR [95% CI] | Z (*P* value) | Heterogeneity of study design | | | Meta-analysis model |
| --- | --- | --- | --- | --- | --- | --- |
|  |  |  | χ^2^ | df (*P* value) | I^2^ (%) |  |
| ***Adiponectin rs3774261 polymorphism*** | | | | | | |
| Adiponectin rs3774261 polymorphism in total population from 2 case-control studies [45, 46] (205 cases and 436 controls) | | | | | | |
| Allelic model (G-allele vs. A-allele) | 1.56 [0.61, 3.99] | 0.93 (0.35) | 12.59 | 1 (<0.001) | 92 | Random |
| Recessive model (GG vs. AG + AA) | 1.47 [0.96, 2.23] | 1.79 (0.07) | 2.41 | 1 (0.12) | 58 | Fixed |
| Dominant model (AG + GG vs. AA) | 2.12 [0.32, 13.84] | 0.79 (0.43) | 18.17 | 1 (<0.001) | 94 | Random |
| Homozygous model (GG vs. AA) | 2.70 [0.36, 20.07] | 0.97 (0.33) | 11.64 | 1 (<0.001) | 91 | Random |
| Heterozygous model (AG vs. AA) | 1.93 [0.30, 12.51] | 0.69 (0.49) | 16.53 | 1 (<0.001) | 94 | Random |

**Abbreviations**: OR, odds ratio; 95% CI, 95% confidence interval.
